# Elevation of plasma tRNA fragments as a promising biomarker for liver fibrosis in nonalcoholic fatty liver disease

**DOI:** 10.1038/s41598-021-85421-0

**Published:** 2021-03-15

**Authors:** Peng Huang, Biao Tu, Hui-jun Liao, Fei-zhou Huang, Zhen-zhou Li, Kuang-ye Zhu, Feng Dai, Huai-zheng Liu, Tian-yi Zhang, Chuan-zheng Sun

**Affiliations:** 1 Department of General Surgery, Xiangya Hospital Central South University, No. 87 Xiangya Road, Changsha, 410008 Hunan People’s Republic of China; 2 Department of General Surgery, Central South University Third Xiangya Hospital, No. 138 Tongzipo Road, Changsha, 410013 Hunan People’s Republic of China; 3 Department of General Surgery, Chenzhou No. 1 People’s Hospital, No. 102 Luojiajing Road, Chenzhou, 423000 Hunan People’s Republic of China; 4 Emergency Department, Central South University Third Xiangya Hospital, No. 138 Tongzipo Road, Changsha, 410013 Hunan People’s Republic of China

**Keywords:** Liver fibrosis, Non-alcoholic fatty liver disease, Non-alcoholic steatohepatitis

## Abstract

Fibrotic tissue remodelling in nonalcoholic fatty liver disease (NAFLD) will probably emerge as the leading cause of end-stage liver disease in the coming decades, but the ability to diagnose liver fibrosis in NAFLD patients noninvasively is limited. The abnormal expression of tRNA-derived small RNA (tsRNA) in plasma provides a novel idea for noninvasive diagnosis of various diseases, however, the relationship between tsRNAs and NAFLD is still unknown. Here, we took advantage of small RNA-Seq technology to profile tsRNAs in NAFLD patients and found the ubiquitous presence of hepatic tsRNAs secreted into circulating blood. Verification in a cohort of 114 patients with NAFLD and 42 patients without NAFLD revealed that three tsRNAs (tRF-Val-CAC-005, tiRNA-His-GTG-001, and tRF-Ala-CGC-006) were significantly elevated in the plasma of NAFLD patients, and the expression level are associated with NAFLD activity score (calculated from 0 to 8) and fibrosis stage (scored from 0 to 4). In mouse models, we further found that increased plasma levels of these three tsRNAs were positively correlated with the degree of liver fibrosis. Our study potentially identifies a new class of NAFLD biomarkers and reveal the possible existence of tsRNAs in the blood that can be used to predict fibrogenesis risk in patients diagnosed with NAFLD.

## Introduction

Nonalcoholic fatty liver disease (NAFLD) is an emerging health problem worldwide due to its growing incidence and prevalence^1^. NAFLD refers to a spectrum ranging from noninflammatory isolated steatosis to nonalcoholic steatohepatitis (NASH), which is characterized by steatosis, necroinflammatory changes, and varying degrees of liver fibrosis^2^. In addition, progressive fibrosis can progress to cirrhosis and hepatocellular carcinoma.

It is well known that hepatocellular carcinoma and cardiovascular complications are life-threatening comorbidities of both NAFLD and NASH^3^. However, the diagnosis of liver fibrosis in NAFLD patients is complex and challenging, as the gold-standard method liver biopsy is a costly and invasive procedure with a high risk of complications^4^. Although clinical trials have shown promising results, no effective medical interventions exist that completely reverse liver fibrosis in NAFLD^5^. Therefore, the rapidly increasing prevalence of NAFLD and of its aggressive form NASH will require novel noninvasive liver fibrosis-forecast approaches to prevent disease progression to advanced fibrosis or cirrhosis and cancer.

The most common scores that combine several clinical parameters are the NAFLD Fibrosis Score (NFS), the Aspartate Transaminase (AST) to Platelet Ratio Index (APRI), and the Fibrosis-4 Score (FIB-4)^5^. However, most biomarkers do not measure fibrinolysis or fibrogenesis directly. Circulating blood-based molecules represent an attractive source of fibrogenesis biomarkers, given the potential for the fast analysis of easy-to-collect samples. Small noncoding RNAs (sncRNAs) have recently emerged as potential biomarkers since changes in miRNA expression profiles, such as miR-122, miR-34a, and miR-192, have been observed at various stages of NAFLD in both human patients and animal models^6^.

tRNA-derived small RNAs (tsRNAs) are novel sncRNAs and tRNA fragments generated from precursor or mature tRNAs with a length of 18–40 nucleotides (nt)^7^. In humans, tRNA fragments are generated by ribonucleases, including Dicer and angiogenin, and participate in many biological processes, including the regulation of gene expression, initiation of stress granule formation and inhibition of protein translation^8^. Recently, tRNA fragments have also been found circulating in the blood and may be treated as ideal candidates for investigation as biomarkers for various diseases, including epilepsy and cancers^9,10^. However, the expressive features and functions of these tRNA fragments in NAFLD remain unknown.

Here, we collected liver tissues and blood samples from 156 patients with gallbladder stones (114 with NAFLD and 42 without (controls)). Then, we identified hepatic tRNA fragments and plasma tRNA fragments in RNA-Seq data from five NAFLD patients and five controls. We found that partial tRNA fragments coexisted in liver tissues and plasma, and 3 specific tRNA fragments were significantly elevated in liver tissues and plasma from NAFLD patients. Further validation in clinical samples and animal models revealed that the plasma levels of these three tsRNAs were positively correlated with the degree of liver fibrosis in NAFLD patients. Together, these data suggest that specific tRNA fragments may constitute a novel class of NAFLD biomarkers that could support the prediction of fibrogenesis risk in patients diagnosed with NAFLD.

## Results

### Patient characteristics

The clinical features of the 156 candidates (114 with NAFLD and 42 without (controls)) are reported in Table 1. The average age of the patients was 45.58 ± 1.91 years, and 73.7% were female. In this study, patient age at the time of sample collection, sex, alanine aminotransferase (ALT), AST, gamma-glutamyl transferase (GGT), alkaline phosphatase (ALP), total bilirubin (Tbil), direct bilirubin (Dbil), fasting blood glucose (FBG), and high-density lipoprotein (HDL) did not differ significantly between the two groups (NAFLD vs control). However, body mass index (BMI), low-density lipoprotein (LDL), triglyceride (TG), total cholesterol (TC), and NAFLD activity score were significantly higher in the NAFLD group than in the control group.

**Table 1:** Demographic and clinical characteristics of subjects.

Factor	NAFLD (N = 114)	Control (N = 42)	p-value
**Demographics**			
Female patients	84 (73.7%)	30 (71.4%)	0.1622^†^
Age (year)	46 ± 2.73	45.16 ± 2.75	0.8292^‡^
BMI (kg/m2)	25.08 ± 0.56	22.78 ± 0.6194	**0.0093**^**‡**^
**Biochemical profile**			
ALT (IU/L)	65.47 ± 25.97	44.42 ± 14.71	0.4851^‡^
AST (IU/L)	63.89 ± 23.87	25.74 ± 3.11	0.1217^‡^
GGT (U/L)	128.2 ± 52.47	66.33 ± 26.9	0.3284^‡^
ALP (U/L)	113 ± 15.31	98.77 ± 12.67	0.4844^‡^
TBil (μmol/L)	13.8 ± 1.988	15.58 ± 2.923	0.6168^‡^
DBil (μmol/L)	5.284 ± 1.37	6.074 ± 1.74	0.7236^‡^
FBG (mmpl/L)	5.044 ± 0.2014	4.705 ± 0.1465	0.1825^‡^
HDL (mmpl/L)	1.15 ± 0.1046	1.33 ± 0.1848	0.4108^‡^
LDL (mmpl/L)	3.445 ± 0.2972	2.22 ± 0.2414	**0.0362**^**‡**^
TG (mmpl/L)	2.201 ± 0.3749	1.309 ± 0.2462	**0.0239**^**‡**^
TC (mmpl/L)	5.4 ± 0.347	4.411 ± 0.2408	**0.0178**^**‡**^

Values presented as mean ± standard deviation or N (column %).

*BMI* body mass index, *ALT* alanine aminotransferase, *AST* aspartate aminotransferase, *GGT* gamma-glutamyl transferase, *ALP* alkaline phosphatase, *Tbil* total bilirubin, *Dbil* direct bilirubin, *FBG* fasting blood glucose, *HDL* high-density lipoprotein, *LDL* low-density lipoprotein, *TG* triglycerides, *TC* total cholesterol, *NAFLD* non-alcoholic fatty liver disease.

p-values: ^†^Pearson’s chi-squared test, ^‡^Student's t‐test.

The histologic features of the NAFLD patients are presented in Table 2. After scored the fibrosis stage of the NAFLD patients, 51 patients were identified with varying degrees of liver fibrosis. Patients with fibrosis had higher grades of steatosis, portal inflammation and ballooning (*p* < 0.001 for all). Besides, the NAFLD activity score was significantly higher in those with fibrosis compared to those without fibrosis (4.53 ± 2.19 vs 2.29 ± 1.17, *p* < 0.001).

**Table 2:** Histologic features of the NAFLD patients.

Factor	Total (N = 114)	No fibrosis (F0) (N = 63)	Fibrosis (F1–3) (N = 51)	p-value
**Steatosis**				**0.000**^**†**^
< 5%	57 (50.0)	45 (71.4)	12 (23.5)	
5–33%	42 (36.8)	18 (28.6)	24 (47.1)	
34–65%	12 (10.5)	0 (0.0)	12 (23.5)	
≥ 66%	3 (2.6)	0 (0.0)	3 (5.9)	
**Portal inflammation (under 20 ×)**				**0.000**^**†**^
None	51 (44.7)	45 (71.4)	6 (11.8)	
< 2	45 (39.5)	18 (28.6)	27 (52.9)	
2–4	9 (7.9)	0 (0.0)	9 (17.6)	
> 4	9 (7.9)	0 (0.0)	9 (17.6)	
**Ballooning**				**0.000**^**†**^
None	27 (23.7)	18 (28.6)	9 (17.6)	
Few	75 (65.8)	45 (71.4)	30 (58.8)	
Many	12 (10.5)	0 (0.0)	12 (23.5)	
**NAS**^**§**^	3.29 ± 2.03	2.29 ± 1.17	4.53 ± 2.19	**0.000**^**‡**^

Values presented as mean ± standard deviation or N (column %).

*NAFLD* non-alcoholic fatty liver disease, *NAS*^*§*^ NAFLD activity score.

Fibrosis was scored as F0, no fibrosis; F1, portal fibrosis without septae; F2, portal fibrosis with septae; F3, numerous septae without cirrhosis; and F4, cirrhosis.

NAFLD patients and non-NAFLD patients.

p-values: ^†^Pearson’s chi-squared test, ^‡^Student's t‐test.

### Partial hepatic tRNA-derived fragments secreted into circulating blood

To comprehensively profile tsRNAs in liver tissues and plasma from patients with NAFLD, small RNA-Seq (< 50 nt) was performed on pooled samples from five NAFLD patients and five controls. Histopathological features of the tissues used for small RNA sequencing are shown in supplemental Fig. 1. A custom tRNA library was used to quantify reads aligning to tRNAs, and only high-quality reads with 14–40 nt insertions were mapped to the human genome and annotated. After further screening, a total of 33 tRNA-derived fragments in liver tissue and 31 in plasma were identified as differentially expressed tsRNAs with fold change filtering (absolute fold change > 2.0), a standard Student’s t-test (*p* < 0.05), and multiple hypothesis testing (FDR < 0.05) (supplemental Table 2 and Table 3).

**Figure 1:**
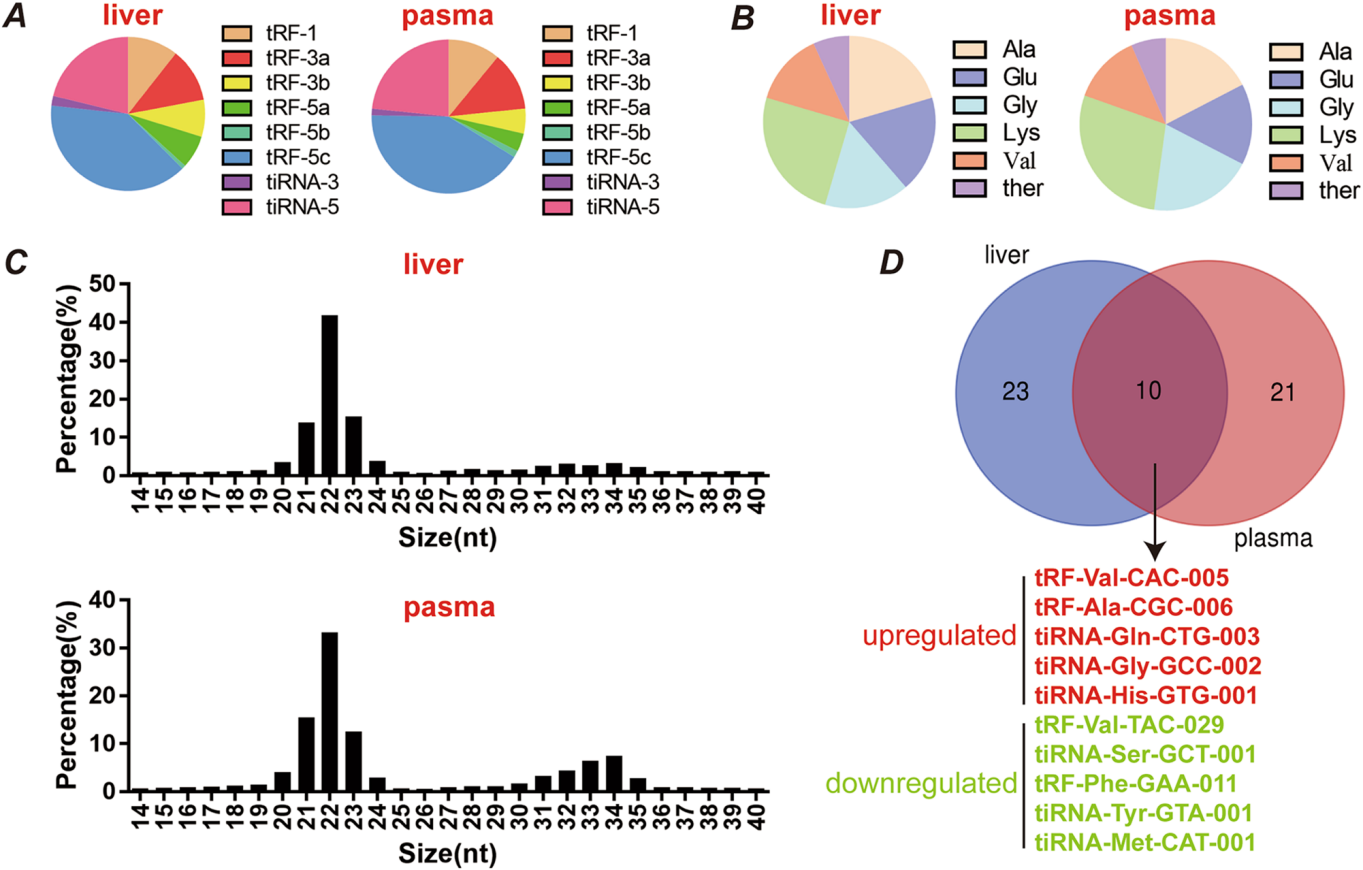
The characteristics of the differentially expressed tsRNAs in liver tissues or plasma are similar between patients with and without NAFLD. **(A)** Percentage of each type of tsRNA; **(B)** percentage of tsRNAs generated from Ala-, Glu-, Gly-, Lys- and Val-tRNAs; **(C)** length distribution of tsRNAs; **(D)** five upregulated and five downregulated tRNA-derived fragments were identified as differentially altered in the liver tissues and plasma of NAFLD patients relative to non-NAFLD patients.

In our results, the percentage of each subtype of differentially expressed tRNA-derived fragments indicated that more than 60% of the fragments were tRF-5c and tiRNA-5 in liver tissues, and the same percentage was observed in plasma (Fig. 1A). Similarly, 75% of the fragments were derived from four tRNAs (Ala-, Glu-, Gly- and Lys-tRNAs) in both liver tissues and plasma (Fig. 1B). Moreover, the large majority of the fragments were mainly 21–23 nt in length and showed one peak in both liver tissues and plasma (Fig. 1C). Thus, the results suggest that tsRNAs can be incorporated into the extracellular environment and secreted into circulating blood. Of note, when comparing the differentially expressed tsRNAs in liver tissues with those in plasma, five upregulated tsRNAs (tRF-Val-CAC-005, tRF-Ala-CGC-006, tiRNA-Gln-CTG-003, tiRNA-Gly-GCC-002, and tiRNA-His-GTG-001) and five downregulated tsRNAs (tRF-Val-TAC-029, tiRNA-Ser-GCT-001, tRF-Phe-GAA-011, tiRNA-Tyr-GTA-001, and tiRNA-Met-CAT-001) coexisted (Fig. 1D) and were chosen for further investigation.

### The levels of three tsRNAs (tRF-Val-CAC-005, tRF-Ala-CGC-006, and tiRNA-His-GTG-001) are elevated in plasma and could serve as potential biomarkers for NAFLD

**Figure 2:**
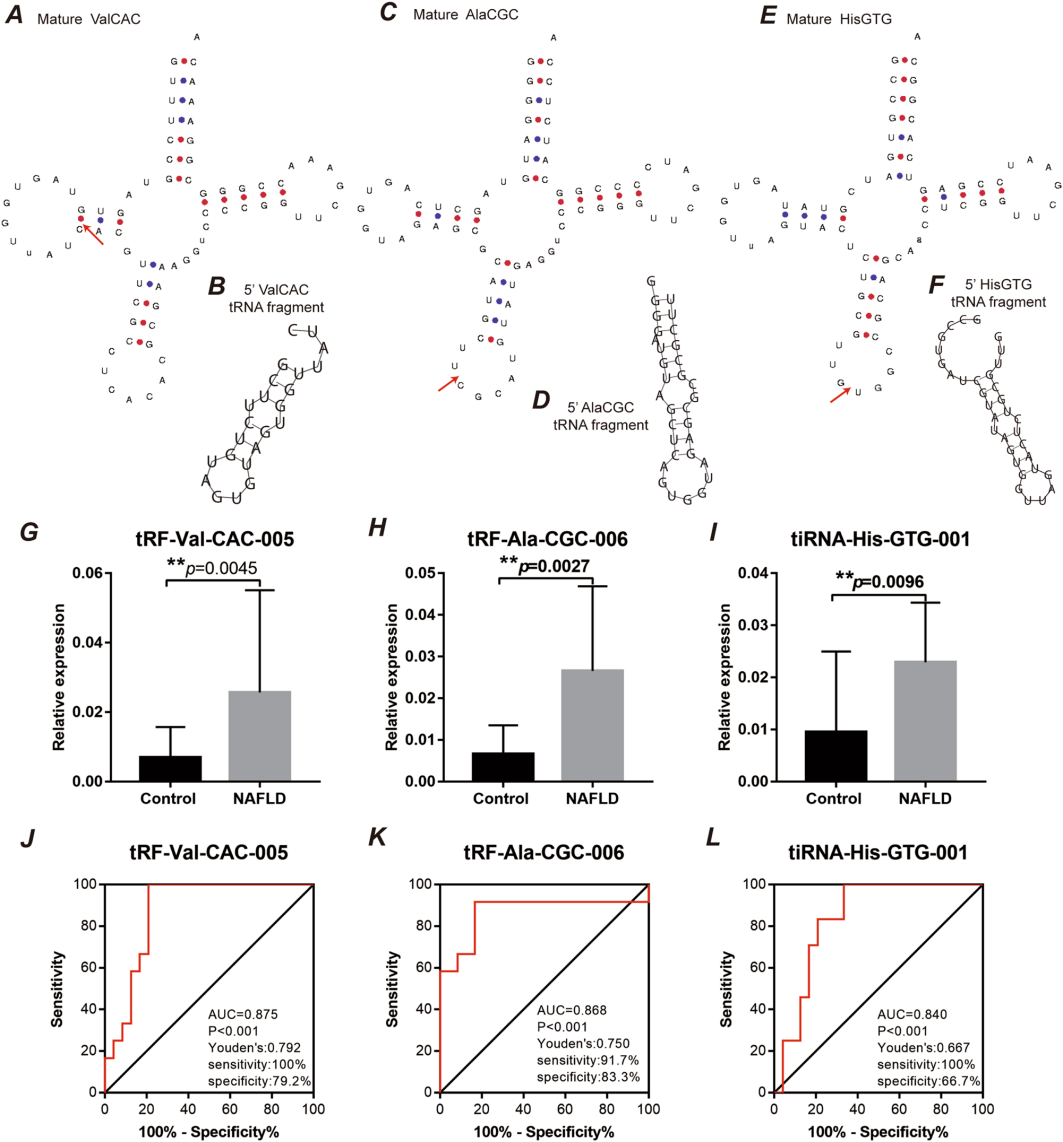
tRNA fragments are elevated in plasma from patients with NAFLD. Blood samples from 114 patients with NAFLD and 42 controls were analysed. **(A,C,E)** Cleavage sites are indicated (red triangle) on the mature tRNA structures (downloaded from GtRNAdb 2.0). **(B,D,F)** Predicted secondary structures of the tRNA fragments. **(G)** tRF-Val-CAC-005, **(H)** tiRNA-His-GTG-001, and **(I)** tRF-Ala-CGC-006 were significantly elevated in the NAFLD samples. Student’s t-test indicated *p* = 0.0045, *p* = 0.0027, and *p* = 0.0096, respectively. **p < 0.01. Data represent the mean ± SEM. ROC curve analysis indicated that **(J)** tRF-Val-CAC-005 had an AUC of 0.875 (*p* < 0.001), **(K)** tiRNA-His-GTG-001 had an AUC of 0.868 (*p* < 0.001), and **(L)** tRF-Ala-CGC-006 had an AUC of 0.840 (*p* < 0.001).

According to the cleavage position on the cloverleaf secondary structure of the derived tRNAs, tRF-Val-CAC-005 was identified as tRF-5b, tRF-Ala-CGC-006 was identified as tRF-5c, and tiRNA-His-GTG-001 identified as was tiRNA-5 (supplemental Fig. 2). We mapped the cleavage site to the predicted secondary structure of each of the tRNAs via GtRNAdb (http://gtrnadb.ucsc.edu/index.html) (Fig. 2A,C,E) and predicted the secondary structures of the tRNA fragments via the RNAfold web server (http://rna.tbi.univie.ac.at//cgi-bin/RNAWebSuite/RNAfold.cgi) (Fig. 2B,D,F).

To explore the potential value of plasma tsRNAs for NAFLD diagnosis, we used qPCR to validate the RNA-Seq data in NAFLD group and non-NAFLD group. We found that only 3 of the ten coexisting tRNA fragments between liver tissues and plasma were significantly elevated in the plasma samples of NAFLD patients (Fig. 2G–I and supplemental Fig. 3), with a highly significant change in tRNA fragment levels between the NAFLD and control groups: tRF-Val-CAC-005, 3.7-fold change (*p* = 0.0045); tRF-Ala-CGC-006, 4.1-fold change (*p* = 0.0027); and tiRNA-His-GTG-001, 2.42-fold change (*p* = 0.0096). Importantly, ROC curve analysis indicated that these three tRNA fragments could distinguish NAFLD and control samples (Fig. 2J–L). tRF-Val-CAC-005 had an AUC of 0.875 (*p* < 0.001, Fig. 2J); tRF-Ala-CGC-006 had an AUC of 0.868 (*p* < 0.001, Fig. 2K); and tiRNA-His-GTG-001 had an AUC of 0.840 (*p* < 0.001, Fig. 2L). Moreover, we applied Youden’s J statistic to determine the optimal cutoff for distinguishing NAFLD and control samples, which indicated that a value of 0.792 was most discriminatory for tRF-Val-CAC-005, with a sensitivity of 100% and a specificity of 79.2% (Fig. 2J); that a value of 0.750 was optimal for tRF-Ala-CGC-006 (Fig. 2K); and that a value of 0.667 performed best for tiRNA-His-GTG-001 (Fig. 2K). These analyses indicate that specific tRNA fragments can discriminate between NAFLD and non-NAFLD samples and may be of use as NAFLD biomarkers.

**Figure 3:**
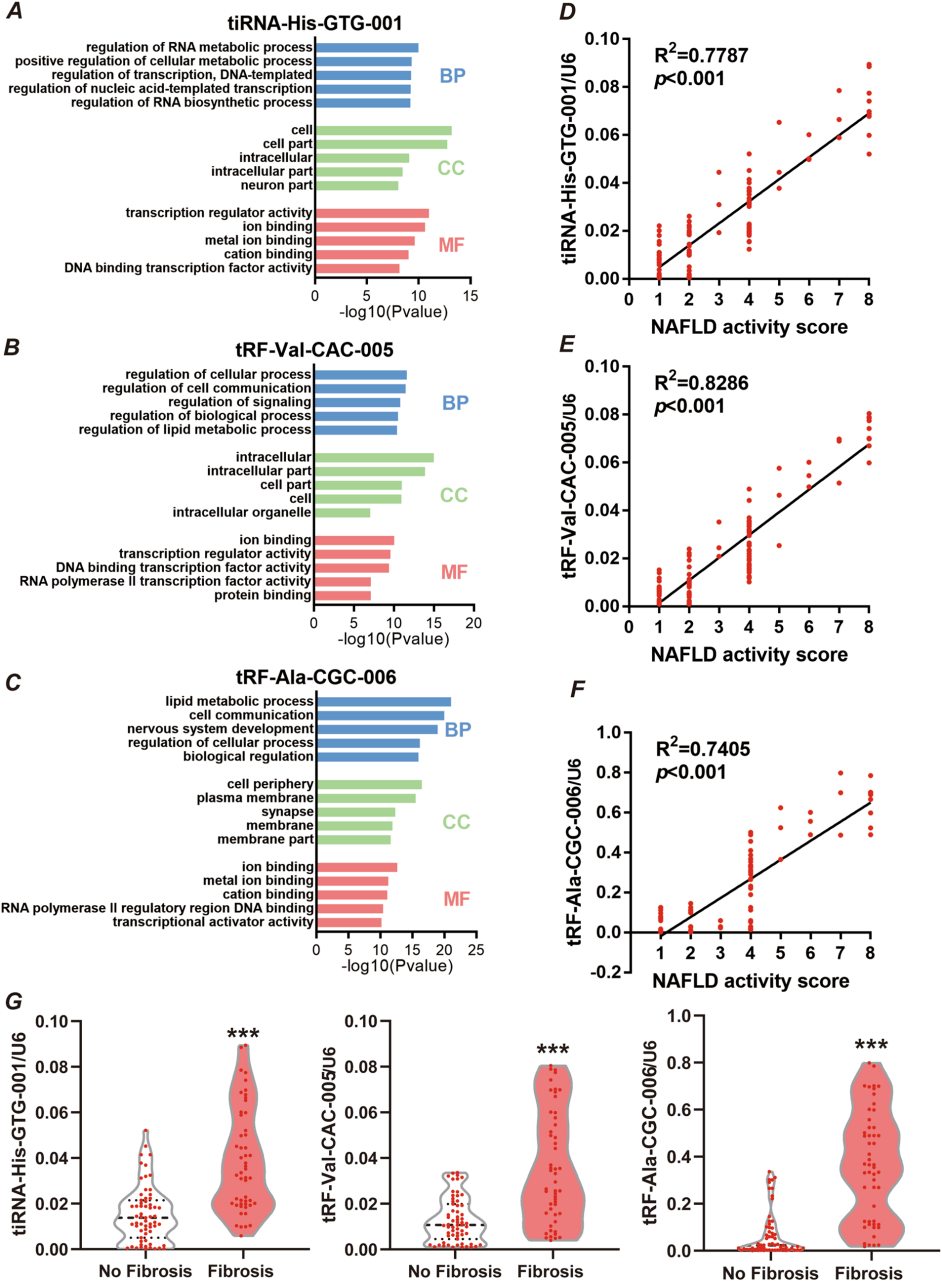
Upregulation of tRNA fragments in plasma correlated with a high NAFLD activity score in patients with NAFLD. The top 5 enriched Gene Ontology (GO) terms in biological process (BP), cellular component (CC), and molecular function (MF) categories for the target genes of **(A)** tRF-Val-CAC-005, **(B)** tiRNA-His-GTG-001, and **(C)** tRF-Ala-CGC-006 are listed. The correlation analyses of the NAFLD activity score and **(D)** tRF-Val-CAC-005, **(E)** tiRNA-His-GTG-001, and **(F)** tRF-Ala-CGC-006 expression in plasma from patients with NAFLD are shown. The NAFLD activity score is plotted on the x-axis, and the tRNA fragment level is normalized to that of U6 on the y-axis, n = 114, R^2^ = 0.7787 (*p* < 0.001), R^2^ = 0.8286 (*p* < 0.0001), R^2^ = 0.7405 (*p* < 0.0001), respectively, Pearson’s r test. **(G)** Violin plot showing the relative expression of tRF-Val-CAC-005, tiRNA-His-GTG-001, and tRF-Ala-CGC-006 in plasma between NAFLD patients without fibrosis (n = 63) and NAFLD patients with any fibrosis (n = 51), ∗  ∗  ∗ *p* < 0.001, Student’s t-test.

### tRF-Val-CAC-005, tRF-Ala-CGC-006, and tiRNA-His-GTG-001 may participate in the progression of NAFLD

As no existing prediction software is able to predict the target genes of tsRNAs, we designed custom Aksomics (Shanghai, China) prediction software combining TargetScan data (http://www.targetscan.org/vert_72/) to obtain target genes. Then, the possible biological functions of the target genes were predicted from Gene Ontology (http://www.geneontology.org). As shown in Fig. 3A–C, tRF-Val-CAC-005, tRF-Ala-CGC-006, and tiRNA-His-GTG-001 mainly participate in the regulation of cellular processes, especially the regulation of lipid metabolic processes, which is consistent with the pathogenic mechanism of NAFLD.

Moreover, a significantly positive correlation between the plasma tsRNA levels (tRF-Val-CAC-005, tRF-Ala-CGC-006, and tiRNA-His-GTG-001) and NAFLD activity score was observed (R^2^ = 0.7787, *p* < 0.001; R^2^ = 0.8286, *p* < 0.001; and R^2^ = 0.7405, *p* < 0.001; respectively) (Fig. 3D–F). In addition, the specific tsRNAs (tRF-Val-CAC-005, tRF-Ala-CGC-006, and tiRNA-His-GTG-001) expression levels in plasma also be found significantly higher in subjects with any fibrosis, significant fibrosis and advanced fibrosis (*p* < 0.001 for all) (Fig. 3G).

### The plasma levels of tRF-Val-CAC-005, tRF-Ala-CGC-006, and tiRNA-His-GTG-001 as predictors of liver fibrosis in NAFLD mouse models

**Figure 4:**
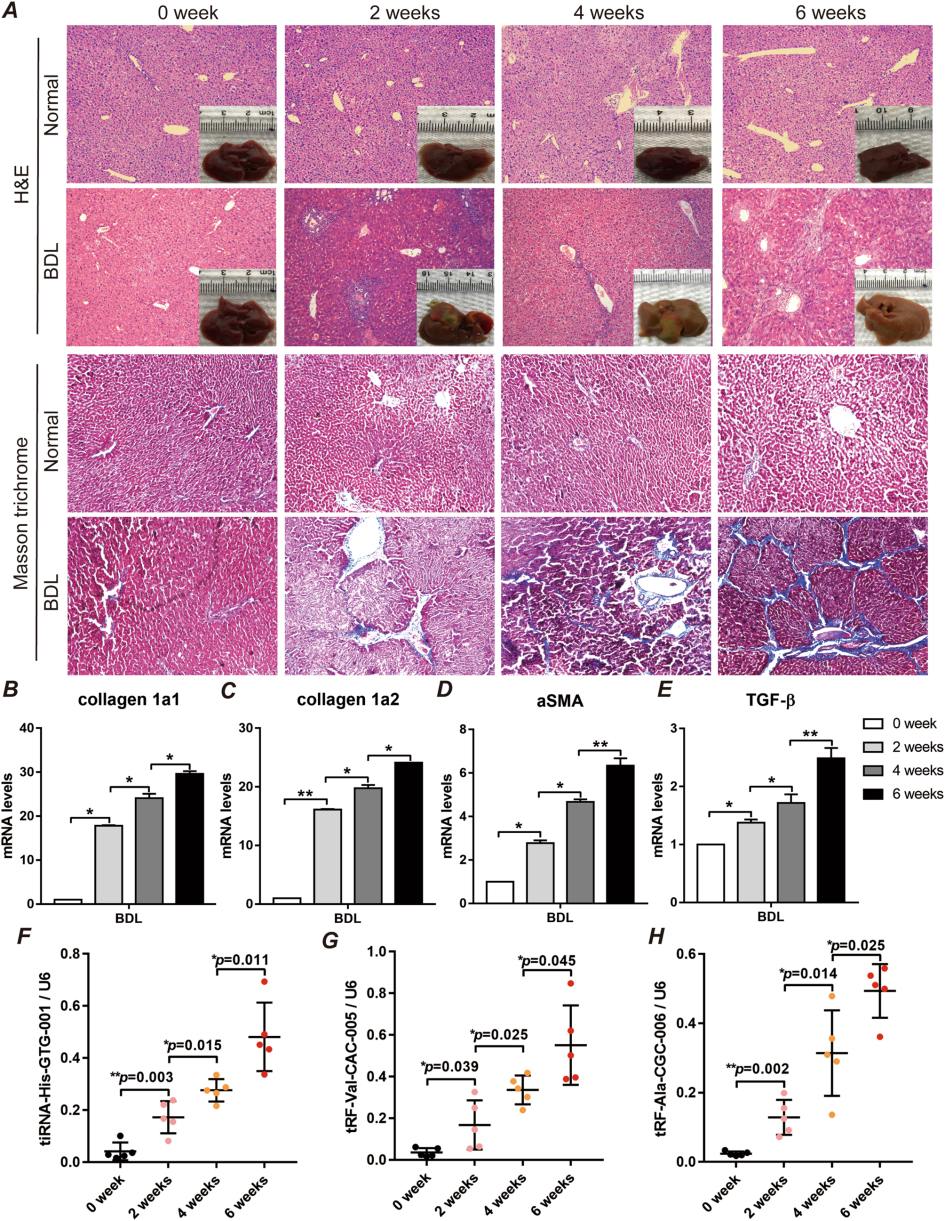
tRNA fragments gradually increased in plasma with the progression of liver fibrosis in the NAFLD mouse model. After being fed an HC diet for 4 weeks, BALB/c mice were subjected to 0, 2, 4, or 6 weeks of BDL to induce liver fibrosis (n = 5/group). **(A)** H&E-stained sections and Masson trichrome-stained sections in representative liver samples, 200 × . **(B–D)** Quantification of the hepatic expression of collagen 1α1, collagen 1α2, αSMA and TGF-β. n = 5, ∗ *p* < 0.05, ∗  ∗ *p* < 0.01, Student’s t-test. **(F–H)** The expression of tRF-Val-CAC-005, tiRNA-His-GTG-001, and tRF-Ala-CGC-006 was tested in plasma by qPCR, n = 5, ∗ *p* < 0.05, ∗  ∗ *p* < 0.01, Student’s t-test.

To explore the potential value of plasma tsRNAs for fibrosis evaluation during NAFLD development, we built an NAFLD mouse model with varying degrees of fibrosis according to our previous report^11^. As the duration of BDL interventions increased, we clearly observed from the pathological point of view that BDL-induced liver fibrosis gradually exacerbated over time (Fig. 4A and supplemental Fig. 4). Moreover, the mRNA expression of collagen 1*α*1, collagen 1α2, αSMA, and TGF-β was significantly promoted as a result of liver fibrosis induced by BDL (Fig. 4B–E). Interestingly, when we further measured the level of plasma tsRNAs in the mice, we found that the plasma levels of tRF-Val-CAC-005, tRF-Ala-CGC-006, and tiRNA-His-GTG-001 were gradually elevated with increasing liver fibrosis (Fig. 4F–H). From our results, we can conclude that the plasma levels of tRF-Val-CAC-005, tRF-Ala-CGC-006, and tiRNA-His-GTG-001 are positively correlated with liver fibrosis.

## Discussion

The present study first demonstrated that tRNA fragment features differ between humans with and without NAFLD. There are currently no reliable biomarkers of NAFLD, especially for the high-risk populations of individuals with advanced NAFLD and liver fibrosis^12^. The ability to forecast fibrogenesis activity would allow patients to regain control over their condition by necessary interventions. Here, we analysed RNA-Seq data from patients with or without NAFLD and verified that 3 tRNA fragments (tRF-Val-CAC-005, tRF-Ala-CGC-006, and tiRNA-His-GTG-001) were elevated in NAFLD plasma samples. We showed that these fragments are expressed by and secreted from hepatocytes and that tRNA fragment levels can be directly used for the diagnosis of NAFLD. Finally, through animal models, we presented a proof-of-concept study indicating that plasma tRNA fragments warrant further investigation as prodromal biomarkers that could be used to predict fibrogenesis risk in NAFLD patients.

As we known, four non-invasive scoring systems including NFS, FIB-4, BARD, and AST to Platelet Ratio Index (APRI) have been developed to identify steatohepatitis and advanced fibrosis in individuals with NAFLD^13,14^. Since those scoring systems were easy to evaluate liver fibrosis in patients with chronic liver disease using routine laboratory parameters, likes ALT, AST, PLT and patient age^15^. However, the accuracy is modest. The limitation of these scores systems is that they incorporate liver enzymes in the models. Since patients with liver enzymes in the normal range can have the full spectrum of liver fibrosis stages, it remains a shortcoming, and liver enzymes are sensitive to age, which can easily lead to a false positive result^16^. In our results, patients with NAFLD also complicated with hepatolithiasis, the AST and ALT were significantly higher in NAFLD group than non-NAFLD group, which means in the case of liver enzyme damage, the use of conventional scoring systems such as FIB-4 and NFS for the estimation of advanced fibrosis is limited. Therefore, exploring novel non-invasive scoring systems to identify steatohepatitis and advanced fibrosis in individuals with NAFLD is necessary.

In recent years, increasing evidence has suggested that differentially expressed tRNA fragments can serve as potential markers of human disease. Marion et al. found that specific tRNA fragments (5′GlyGCC, 5′AlaTGC, and 5′GluCTC) in plasma are associated with epilepsy and that elevated tRNA fragments forecast seizure risk in patients with epilepsy^9^. Recently, Dhahbi et al. reported that tsRNAs in serum circulated at different levels in breast cancer patients and healthy individuals^17^. Further study indicated that special tRNA fragments, such as tRF-30-JZOYJE22RR33 and tRF-27-ZDXPHO53KSN, are involved in trastuzumab resistance in breast cancer^18^. In our ROC curve analysis, the plasma levels of tRF-Val-CAC-005, tRF-Ala-CGC-006, and tiRNA-His-GTG-001 were associated with NAFLD (Fig. 2J–L). An important significance of the present study is that our research complements new valuable evidence to understand the role of tRNA fragments in human disease.

An increasing number of studies have revealed that ncRNAs may play important regulatory roles in NAFLD initiation and progression. However, the exploration of the mechanism of tRNA fragment-mediated disease progression is still in its infancy^19^. The latest research suggests that different types of tRNA-derived fragments with a variety of different functions function similarly to miRNAs^20,21^. We previously reported that miR-21 was upregulated in free fatty acid (FFA)-challenged HepG2 cells and played an important role in the process of lipogenesis^22^. Here, through functional prediction, we found that specific tRNA fragments (AStRF-Val-CAC-005, tRF-Ala-CGC-006, and tiRNA-His-GTG-001) with diagnostic value for NAFLD may participate in the NAFLD process by regulating lipid metabolism, which is consistent with our previously reported role of RNA in NAFLD^22^.

We next validated the potential value of special tRNA fragments in plasma to predict NAFLD liver fibrosis in a mouse model. Liver fibrosis induced by BDL with a high-fat diet is commonly used to establish an advanced NAFLD model^23^. We also used the expression of collagen 1α1, collagen 1α2, αSMA, and TGFβ to evaluate the degree of liver fibrosis in the mice as previously reported^23^. The mRNA expression of collagen 1α1, collagen 1α2, αSMA, and TGFβ was significantly promoted as a result of the development of BDL-induced liver fibrosis and was more elevated with the length of intervention. Interestingly, the plasma levels of tRF-Val-CAC-005, tRF-Ala-CGC-006, and tiRNA-His-GTG-001 gradually increased in the mice and were consistent with the trend of increased liver fibrosis, which strongly suggests the potential value of these three tsRNAs in predicting liver fibrosis in NAFLD.

We acknowledge the following limitations of this study. This is a single-centre study performed in a centre with expertise in the clinical investigation of NAFLD, and the generalizability of the findings in other clinical settings remains to be established. Further multi-centre studies including a larger number of individuals from diverse geographical origins are needed to validate the clinical utility and applicability of our findings to detect fibrogenesis in NAFLD. Moreover, the function and specific mechanism of tsRNAs in the NAFLD process need further confirmation. In addition, despite the detection of tRNA fragments in resected liver tissues of NAFLD patients and mouse models, we cannot exclude the possibility that the tRNA fragments we detected in the patients’ plasma may have originated in the gallbladder due to cholecystitis, as tRNA cleavage has been identified in response to infection and ischaemia^24,25^. Here, a common contradiction exists: it is not easy to obtain enough liver tissue for tsRNA sequencing from patients just diagnosed with NAFLD, although liver tissue can be obtained by invasive liver biopsy in NAFLD patients.

In summary, we comprehensively analysed tRNA-derived fragments in NAFLD patients and identified tRF-Val-CAC-005, tRF-Ala-CGC-006, and tiRNA-His-GTG-001 as potential biomarkers for NAFLD. The plasma levels of tRF-Val-CAC-005, tRF-Ala-CGC-006, and tiRNA-His-GTG-001 could be used to predict liver fibrogenesis risk. We believe that the results of our study could provide a basis for the further exploration of the biological functions of these novel tRNA-derived fragments in the diagnosis and management of NAFLD patients.

## Methods

### Study approval

This study was approved by the Ethics Committee of the Third Xiangya Hospital of Central South University and conducted according to the principles expressed in the Declaration of Helsinki (2016-S090). In addition, written informed consent was obtained from all patients. The animal work was approved by the Research Ethical Committee of Laboratory Animal Center, Xiangya Medical School, Central South University.

### Human liver tissues and blood samples from NAFLD patients and non-NAFLD group

A total of 156 patients (age range, 22 to 65 years) initially treated in the general surgery department of our institution between June 2015 and December 2019 were recruited for the study. Of these patients, 114 had NAFLD and 42 did not. All patients underwent partial liver resection according to relevant treatment guidelines, as 114 NAFLD patients combined with hepatolithiasis and 42 non-NAFLD patients have Grade III or IV liver injury^26,27^. The details of the demographic and clinical characteristics of the subjects are shown in Table 1. NAFLD activity scores (0–8) and fibrosis stage (0–4) were scored by pathological examinations independently by two senior pathologists according to a NAFLD activity scoring (NAS) system previously reported^28^.

A 10 ml blood sample was taken on admission, plasma was prepared within 1 h of collection by centrifuging (1300×*g*, 10 min, 4 °C) and stored at − 80 °C. Besides, approximately 50–100 mg liver samples were collected after patients underwent partial liver resection and frozen in liquid nitrogen immediately for use in the study.

### Small RNA sequencing

Small RNA seq (< 50 nt) was performed on pooled plasma and liver tissue from 5 patients with histopathologically confirmed NAFLD and 5 patients without NAFLD. Total RNA was extracted from liver or plasma using TRIzol LS Reagent (Invitrogen, USA). The small RNA sequencing library was prepared with a NEXTflex Small RNA-Seq Kit v3 (BIOO SCIENTIFIC, USA) following the manufacturer’s protocol and sequenced on an Illumina X Ten sequencing platform (Aksomics, China).

### Animal models

Forty-eight healthy male (8-week-old) wild-type BALB/c mice were randomly separated into eight experimental groups (n = 6 per group) and fed a high-cholesterol (HC) (1% wt/wt) diet (TD 92181) for 4 weeks, and then underwent bile duct ligation (BDL) for 0, 2, 4, or 6 weeks according to our previous report^11^. At the termination of dosing, blood was collected through the eyeball method. In addition, liver tissues were collected, and a portion was immediately frozen in liquid nitrogen for RNA analysis. The remaining liver tissues were placed in 10% neutral buffered formalin for histology. The mice were treated in accordance with ethical requirements for laboratory animal care. This study was carried out in compliance with the ARRIVE guidelines^29^.

### Reverse transcription and quantitative real-time PCR

Plasma preparation and RNA extraction were performed according to a previous report^9^. Total RNA was subjected to cDNA synthesis by M-MLV Reverse Transcriptase (Invitrogen, USA), and qPCR was performed with SYBR Premix Ex Taq (Takara Bio, China) using a StepOne Plus real-time PCR system (Applied Biosystems). Based on the reported literature, U6 was chosen as an internal control for tsRNA quantification in plasma^30^. The relative expression levels were calculated via the 2^-∆∆Ct^ method^31,32^. The primers for RT and qPCR are listed in supplemental Table 1.

### Statistical analysis

Statistical analysis was performed in GraphPad Prism 7.0 or SPSS 22.0. Data are presented as the fold change relative to control samples. The results are presented as the mean ± SD, and the data were subjected to Pearson’s chi-squared test or Student’s t-test. For all analyses, a p-value less than 0.05 was considered significant. Receiver operating characteristic (ROC) curve analysis was performed in SPSS to determine the area under the curve (AUC), and Youden’s J statistic was used to identify the optimal discriminatory tsRNA level.

## Supplementary Information

Supplementary Information 1.

## Abbreviations

NAFLD: Nonalcoholic fatty liver disease
NASH: Nonalcoholic steatohepatitis
NFS: Fibrosis score
AST: Aspartate transaminase
APRI: Platelet ratio index
FIB-4: The fibrosis-4 score
sncRNAs: Novel small non-coding RNAs
tsRNAs: TRNA-derived small RNAs
nt: Nucleotides
HC: High-cholesterol
BDL: Bile duct ligation
ROC: Receiver operating characteristic
AUC: Area under the curve
ALT: Alanine aminotransferase
GGT: Gamma-glutamyl transferase
ALP: Alkaline phosphatase
Tbil: Total bilirubin
Dbil: Direct bilirubin
FBG: Fasting blood glucose
HDL: High-density lipoprotein
BMI: Body mass index
LDL: Low-density lipoprotein
TG: Triglycerides
TC: Total cholesterol
FFA: Free fatty acid
