# Supplementary material for: Elevation of plasma tRNA fragments as a promising biomarker for liver fibrosis in nonalcoholic fatty liver disease
Source: Sci Rep. 2021 Mar 15;11:5886. doi: 10.1038/s41598-021-85421-0 (PMC7961013; doi:10.1038/s41598-021-85421-0)
Supplement: Supplementary file 1 — Supplementary Information 1. [file 41598_2021_85421_MOESM1_ESM.docx]

Supplemental materials

# Elevation of Plasma tRNA Fragments as a Promising Biomarker for Liver Fibrosis in Nonalcoholic Fatty Liver Disease

Peng Huang^1^, Biao Tu^2^, Hui-jun Liao^3^, Fei-zhou Huang^2^, Zhen-zhou Li^4^, Kuang-ye Zhu^2^, Feng Dai^2^, Huai-zheng Liu^4^, Tian-yi Zhang^4^, Chuan-zheng Sun^4§^

^1^Department of general surgery, Xiangya Hospital Central South University, No.87 Xiangya Road, Changsha 410008, Hunan, P.R. China

^2^Department of general surgery, Central South University Third Xiangya Hospital, No.138 Tongzipo Road, Changsha 410013, Hunan, P.R. China

^3^Department of general surgery, Chenzhou No.1 People's Hospital, No.102 Luojiajing Road, Chenzhou 423000, Hunan, P.R. China

^4^Emergency Department, Central South University Third Xiangya Hospital, No.138 Tongzipo Road, Changsha 410013, Hunan, P.R. China

^§^Corresponding to **Chuan-zheng Sun**, MD., Emergency Department, Central South University Third Xiangya Hospital, No.138 Tongzipo Road, Changsha 410013, Hunan, P.R. China. Tel: +86 13574841516, email: sunchuanzheng@csu.edu.cn

| **Table 1. Primers for Reverse Transcription and Quantitative PCR** | | |
| --- | --- | --- |
| Gene name | Primer sequence | |
| U6 | forward | GCTTCGGCAGCACATATACTAAAAT |
|  | reverse | CGCTTCACGAATTTGCGTGTCAT |
| tRF-Val-CAC-005 | forward | TTCTACAGTCCGACGATCGCT |
|  | reverse | TGCTCTTCCGATCTGATAACCA |
| tRF-Ala-CGC-006 | forward | GATCGGGGATGTAGCTCAGTG |
|  | reverse | TGTGCTCTTCCGATCTAAGCG |
| tiRNA-Gln-CTG-003 | forward | CGATCGGTTCCATGGTGTAAT |
|  | reverse | CGTGTGCTCTTCCGATCTGAG |
| tiRNA-Gly-GCC-002 | forward | ACAGTCCGACGATCGCATG |
|  | reverse | GTGCTCTTCCGATCTCAGGC |
| tiRNA-His-GTG-001 | forward | CGCCGTGATCGTATAGTGGTT |
|  | reverse | TGTGCTCTTCCGATCTCAACG |
| tRF-Val-TAC-029 | forward | TCCGACGATCCAGAGTGTAGC |
|  | reverse | CTTCCGATCTTGGGTGCTTT |
| tiRNA-Ser-GCT-001 | forward | GACGATCGAGAAAGCTCACAAG |
|  | reverse | GACGTGTGCTCTTCCGATCTC |
| tRF-Phe-GAA-011 | forward | AGTCCGACGATCCACATCAC |
|  | reverse | GCTCTTCCGATCTTGGTGTT |
| tiRNA-Tyr-GTA-001 | forward | CGGTAAAATGGCTGAGTGAAG |
|  | reverse | GTGCTCTTCCGATCTCAGTCC |
| tiRNA-Met-CAT-001 | forward | TAAGGTCAGCTAAATAAGCTATCG |
|  | reverse | GTGTGCTCTTCCGATCTGG |


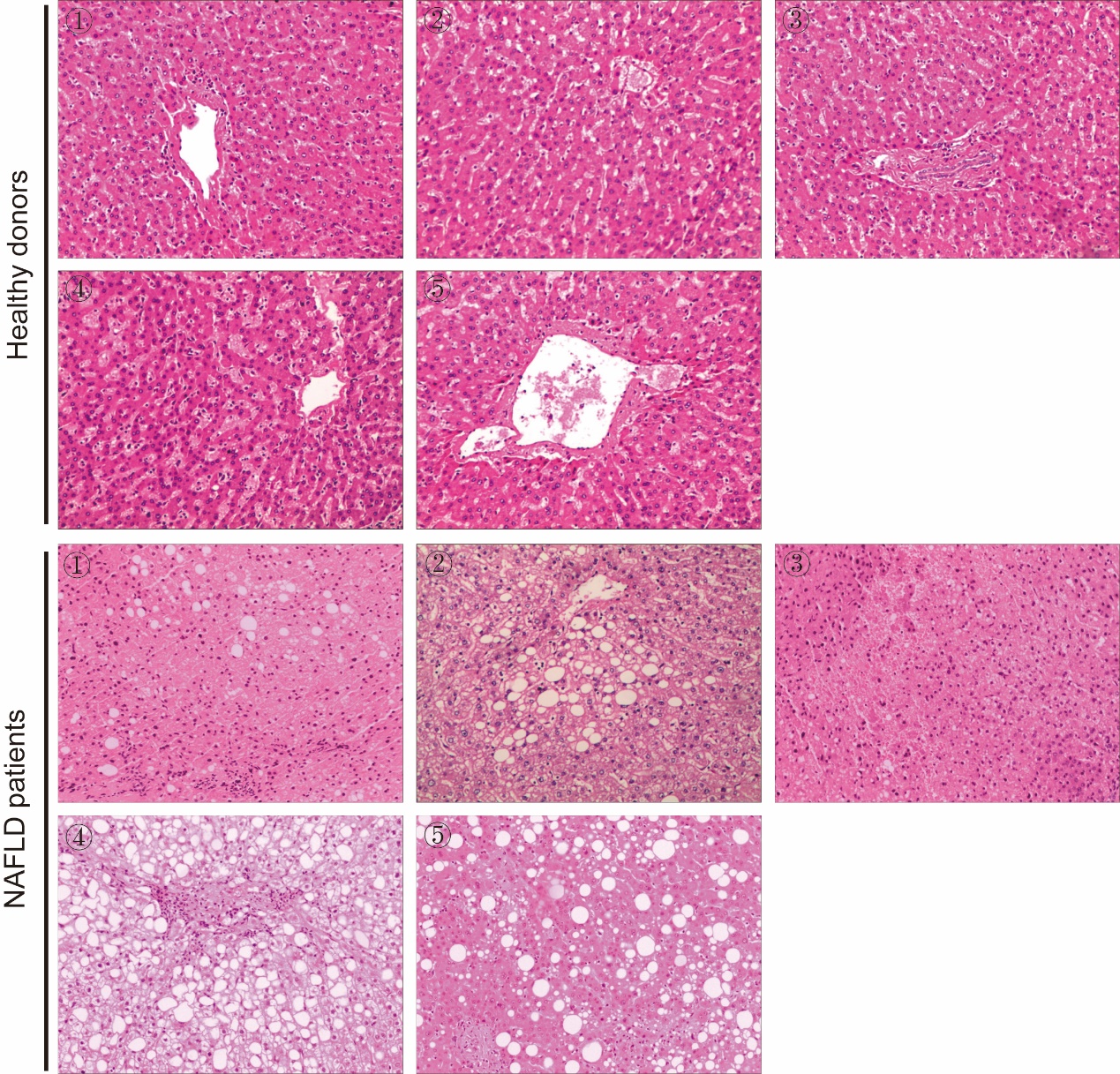


Figure 1. H&E-stained sections in liver samples of healthy controls and NAFLD patients whose tissues were used for small RNA sequencing.


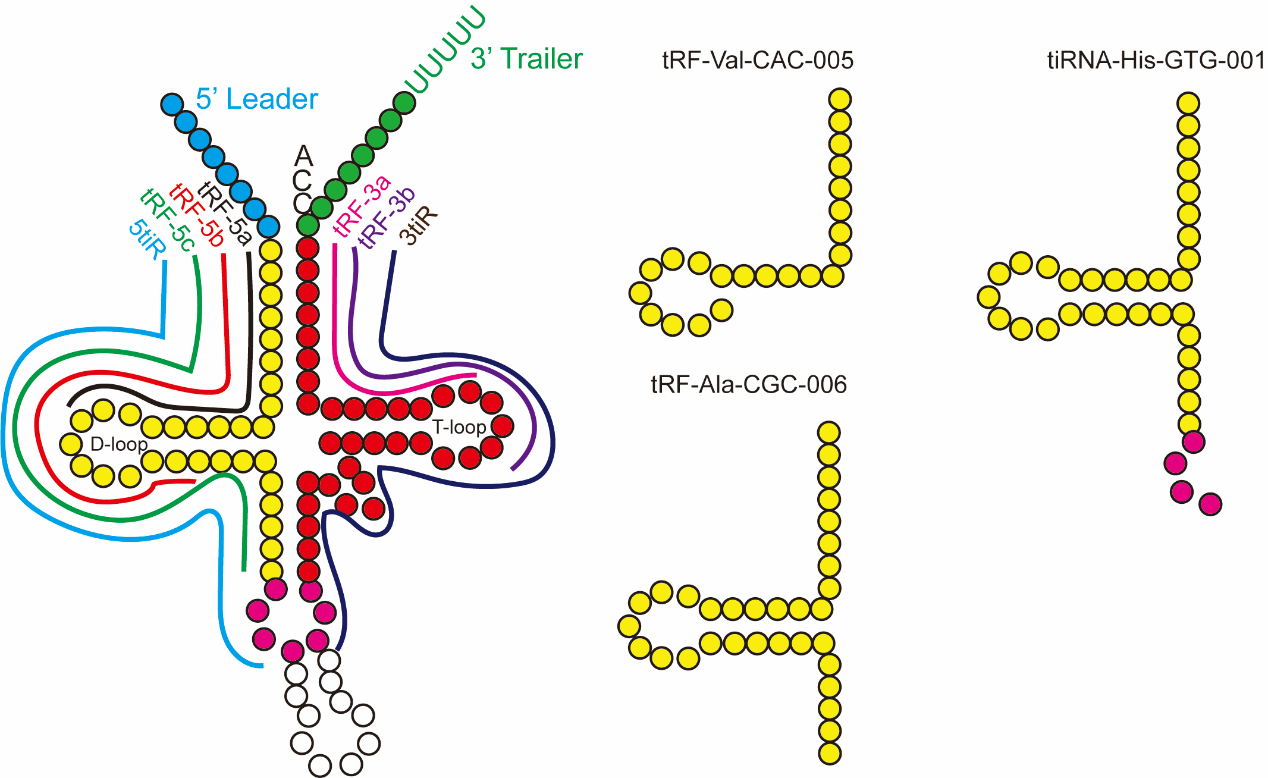


Figure 2. Classification of tsRNAs generated from mature tRNAs.


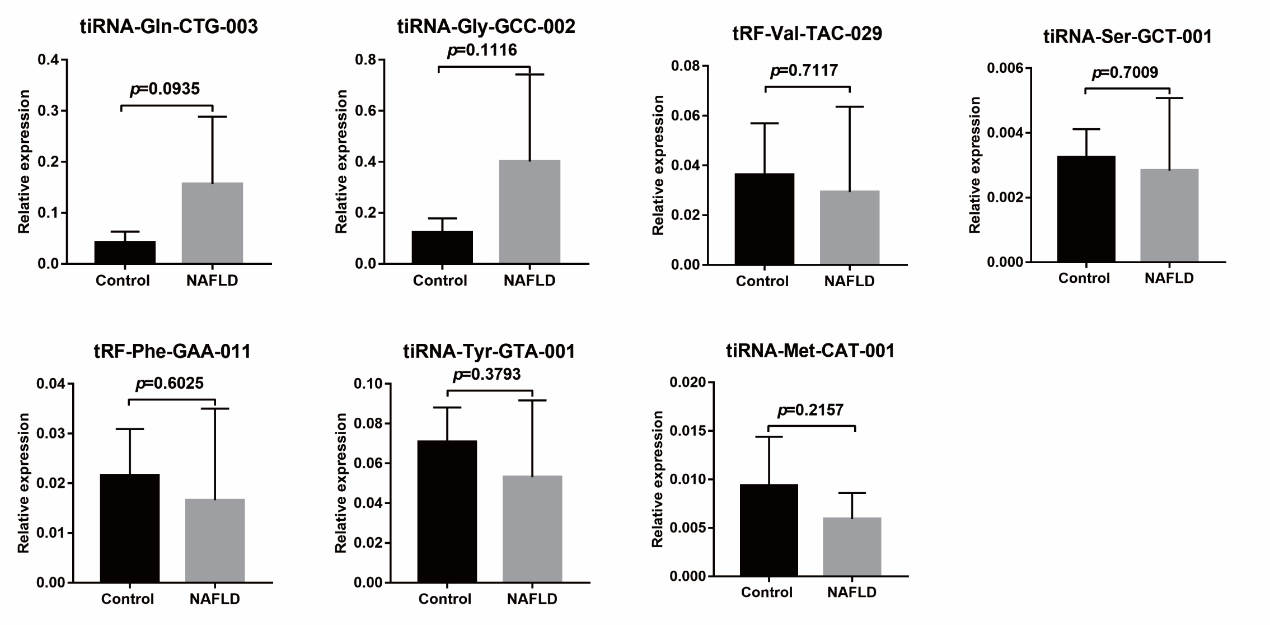


Figure 3. Quantification of the expression of tiRNA-Gln-CTG-003, tiRNA-Gly-GCC-002, tRF-Val-TAC-029, tiRNA-Ser-GCT-001, tRF-Phe-GAA-011, tiRNA-Tyr-GTA-001, and tiRNA-Met-CAT-001 in plasma by qPCR, n (NAFLD) =114, n (Control) =42, Student’s t-test.


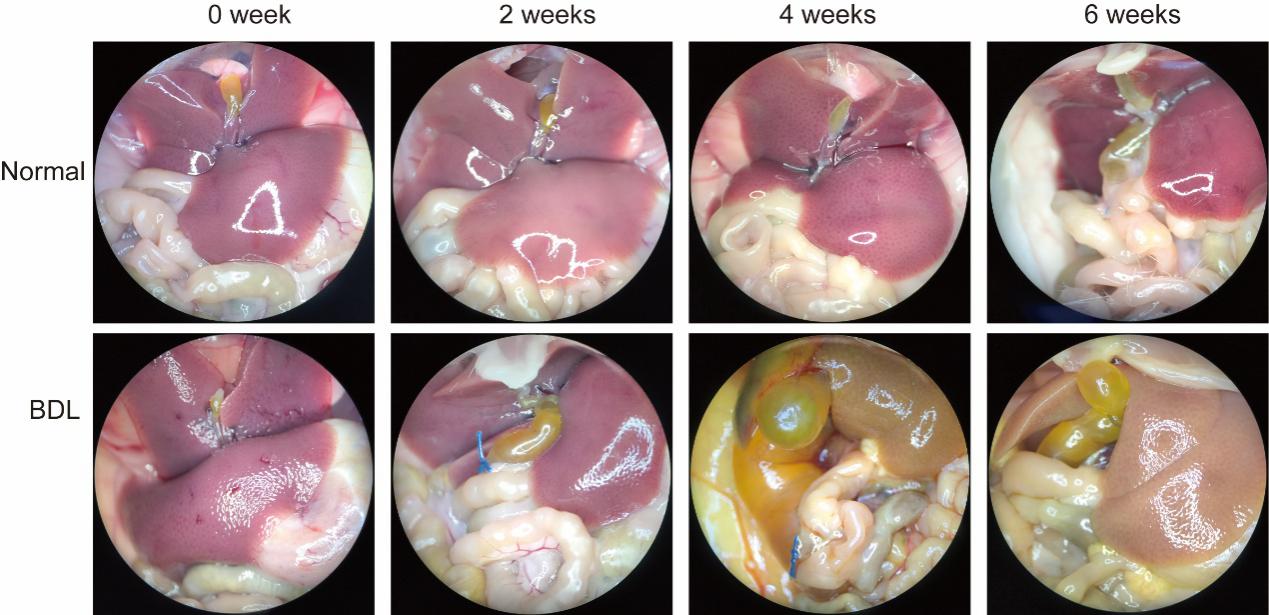


Figure 4. The gross of the changes in mouse livers between normal and BDL groups. The mice were anaesthetized at 0, 2, 4, and 6 weeks, and the appearance of the liver and changes in surrounding organs were observed under a microscope after surgical exposure of the liver.

| Table 2. List of 33 differentially expressed tsRNAs in liver tissues between NAFLD patients and non-NAFLD patients. | | | | | |
| --- | --- | --- | --- | --- | --- |
| **tsRNA-ID** | **tsRNA sequence** | **tRNA gene** | **tsRNA type** | **Log2FC** | **p_value** |
| tRF-His-GTG-028 | GCCATGATCGTATAGTGGTTAGTACTCTGCGC | His-GTG | tRF-5c | 3.23 | 0.009 |
| tiRNA-His-GTG-001 | GCCGTGATCGTATAGTGGTTAGTACTCTGCGTTG | His-GTG | tiRNA-5 | 3.02 | 0.014 |
| tiRNA-Glu-TTC-001 | TCCCACATGGTCTAGCGGTTAGGATTCCTGGTTT | Glu-TTC | tiRNA-5 | 2.99 | 0.013 |
| tRF-Val-CAC-005 | GCTTCTGTAGTGTAGTGGTTATC | Val-CAC | tRF-5b | 2.88 | 0.009 |
| tRF-Ala-AGC-008 | GGGGATGTAGCTCAGTGGTAGAGCGCATGCTT | Ala-AGC | tRF-5c | 2.78 | 0.023 |
| tiRNA-Gln-CTG-002 | GGTTCCATGGTGTAATGGTGAGCACTCTGGACTC | Gln-CTG | tiRNA-5 | 2.65 | 0.015 |
| tRF-Ala-CGC-006 | GGGGATGTAGCTCAGTGGTAGAGCGCGCGCTT | Ala-CGC | tRF-5c | 2.55 | 0.015 |
| tiRNA-Gly-GCC-003 | GCATTGGTGGTTCAGTGGTAGAATTCTCGCCTG | Gly-GCC | tiRNA-5 | 2.53 | 0.012 |
| tiRNA-Gln-CTG-003 | GGTTCCATGGTGTAATGGTTAGCACTCTGGACTC | Gln-CTG | tiRNA-5 | 2.53 | 0.017 |
| tiRNA-Gly-GCC-002 | GCATGGGTGGTTCAGTGGTAGAATTCTCGCCTG | Gly-GCC | tiRNA-5 | 2.53 | 0.013 |
| tRF-Val-CAC-008 | GCTTCTGTAGTGTAGTGGTTATCACGTTC | Val-CAC | tRF-5c | 2.39 | 0.029 |
| tRF-Ala-CGC-020 | GGGGATGTAGCTCAGTGGTAGAGCGCGCGC | Ala-CGC | tRF-5c | 2.25 | 0.038 |
| tRF-Gly-CCC-005 | GCATTGGTGGTTCAATGGTAGAATTCTCGCCT | Gly-CCC | tRF-5c | 2.19 | 0.034 |
| tRF-Gly-GCC-011 | GCATGGGTGGTTCAGTGGTAGAATTCTCGCCT | Gly-GCC | tRF-5c | 2.13 | 0.033 |
| tRF-Gly-CCC-014 | GCATTGGTGGTTCAGTGGTAGAATTCTCGCCT | Gly-CCC | tRF-5c | 2.10 | 0.032 |
| tiRNA-Lys-CTT-002 | GCCCGGCTAGCTCAGTCGGTAGAGCATGAGACTC | Lys-CTT | tiRNA-5 | 2.06 | 0.022 |
| tRF-Lys-CTT-004 | GCCCGGCTAGCTCAGTCGGTAGAGCATG | Lys-CTT | tRF-5c | 1.99 | 0.046 |
| tRF-Leu-TAG-006 | ACTTTTAAAGGATAAC | Leu-TAG | tRF-5a | -3.73 | 0.004 |
| tRF-Phe-GAA-011 | CACATCACCCCATAAACACCA | Phe-GAA | tRF-3b | -2.96 | 0.001 |
| tRF-Val-CAC-016 | AAGTGGTTCCCGTTT | Val-CAC | tRF-1 | -2.78 | 0.005 |
| tRF-Val-TAC-005 | CAGAGTGTAGCTTAAC | Val-TAC | tRF-5a | -2.58 | 0.001 |
| tRF-Lys-TTT-024 | CACTGTAAAGCTAACT | Lys-TTT | tRF-5a | -2.31 | 0.004 |
| tRF-Phe-GAA-031 | TCACCCCATAAACACCA | Phe-GAA | tRF-3a | -2.29 | 0.002 |
| tiRNA-Met-CAT-001 | AGTAAGGTCAGCTAAATAAGCTATCGGGCCC | Met-CAT | tiRNA-5 | -2.28 | 0.000 |
| tiRNA-Pro-TGG-001 | CAGAGAATAGTTTAAATTAGAATCTTAGCTTT | Pro-TGG | tiRNA-5 | -2.04 | 0.001 |
| tRF-Val-CAC-017 | AAGTGGTTCCCGTTTT | Val-CAC | tRF-1 | -1.82 | 0.043 |
| tRF-Glu-TTC-033 | ATGATGTATGCTTTGT | Glu-TTC | tRF-1 | -1.76 | 0.009 |
| tiRNA-Ser-GCT-001 | GAGAAAGCTCACAAGAACTG | Ser-GCT | tiRNA-5 | -1.59 | 0.005 |
| tiRNA-Tyr-GTA-001 | GGTAAAATGGCTGAGTGAAGCATTGGACTG | Tyr-GTA | tiRNA-5 | -1.59 | 0.009 |
| tRF-Phe-GAA-007 | ACATCACCCCATAAACACCA | Phe-GAA | tRF-3b | -1.39 | 0.026 |
| tiRNA-Leu-TAG-002 | ACTTTTAAAGGATAACAGCTATCCATTGGTCTT | Leu-TAG | tiRNA-5 | -1.32 | 0.030 |
| tRF-Leu-CAA-002 | GTCAGGATGGCCGAG | Leu-CAA | tRF-5a | -1.16 | 0.025 |
| tRF-Val-TAC-029 | CAGAGTGTAGCTTAACACAAAGCACCCA | Val-TAC | tRF-5c | -1.12 | 0.041 |
| FC: fold change | | | | | |

| Table 3. List of 31 differentially expressed tsRNAs in plasma between NAFLD patients and non-NAFLD patients. | | | | | |
| --- | --- | --- | --- | --- | --- |
| **tsRNA-ID** | **tsRNA sequence** | **tRNA gene** | **tsRNA type** | **log2FC** | **p_value** |
| tiRNA-Gln-TTG-002 | GGCCCCATGGTGTAATGGTTAGCACTCTGGACTT | Gln-TTG | tiRNA-5 | 4.82 | 0.002 |
| tiRNA-His-GTG-001 | GCCGTGATCGTATAGTGGTTAGTACTCTGCGTTG | His-GTG | tiRNA-5 | 3.02 | 0.014 |
| tiRNA-Gly-GCC-001 | GCATAGGTGGTTCAGTGGTAGAATTCTTGCCTG | Gly-GCC | tiRNA-5 | 2.92 | 0.009 |
| tRF-Val-CAC-005 | GCTTCTGTAGTGTAGTGGTTATC | Val-CAC | tRF-5b | 2.88 | 0.009 |
| tRF-His-GTG-009 | GCCGTGATCGTATAGTGGTTAGTACTCTGCGT | His-GTG | tRF-5c | 2.72 | 0.016 |
| tiRNA-Ala-CGC-002 | GGGGATGTAGCTCAGTGGTAGAGCGCATGCTTC | Ala-CGC | tiRNA-5 | 2.63 | 0.020 |
| tRF-Ala-CGC-006 | GGGGATGTAGCTCAGTGGTAGAGCGCGCGCTT | Ala-CGC | tRF-5c | 2.55 | 0.015 |
| tiRNA-Gln-CTG-003 | GGTTCCATGGTGTAATGGTTAGCACTCTGGACTC | Gln-CTG | tiRNA-5 | 2.53 | 0.016 |
| tiRNA-Gly-GCC-002 | GCATGGGTGGTTCAGTGGTAGAATTCTCGCCTG | Gly-GCC | tiRNA-5 | 2.53 | 0.014 |
| tiRNA-Gln-TTG-003 | GGTCCCATGGTGTAATGGTTAGCACTCTGGACTT | Gln-TTG | tiRNA-5 | 2.26 | 0.048 |
| tiRNA-Pro-TGG-003 | GGCTCGTTGGTCTAGGGGTATGATTCTCGGTTT | Pro-TGG | tiRNA-5 | 2.20 | 0.043 |
| tRF-Gly-GCC-005 | GCATAGGTGGTTCAGTGGTAGAATTCTTGCCT | Gly-GCC | tRF-5c | 2.16 | 0.039 |
| tiRNA-Gly-CCC-002 | GCATTGGTGGTTCAGTGGTAGAATTCTCGCCTC | Gly-CCC | tiRNA-5 | 2.09 | 0.044 |
| tiRNA-Ala-CGC-003 | GGGGATGTAGCTCAGTGGTAGAGCGCGCGCTTC | Ala-CGC | tiRNA-5 | 2.08 | 0.042 |
| tRF-Phe-GAA-021 | GTTTATGTAGCTTACC | Phe-GAA | tRF-5a | -3.55 | 0.001 |
| tRF-Ile-GAT-006 | AGAAATATGTCTGAT | Ile-GAT | tRF-5a | -3.43 | 0.001 |
| tRF-Phe-GAA-012 | CATCACCCCATAAACACCA | Phe-GAA | tRF-3b | -3.23 | 0.001 |
| tRF-Val-CAC-023 | AAGTGGTTCCTGTT | Val-CAC | tRF-1 | -3.21 | 0.005 |
| tRF-Phe-GAA-011 | CACATCACCCCATAAACACCA | Phe-GAA | tRF-3b | -2.96 | 0.003 |
| tRF-Phe-GAA-022 | GTTTATGTAGCTTACCTCCTCA | Phe-GAA | tRF-5b | -2.46 | 0.041 |
| tRF-Trp-TCA-005 | ATACTTAATTTCTGCCA | Trp-TCA | tRF-3a | -2.39 | 0.007 |
| tiRNA-Met-CAT-001 | AGTAAGGTCAGCTAAATAAGCTATCGGGCCC | Met-CAT | tiRNA-5 | -2.28 | 0.001 |
| tRF-Phe-GAA-030 | TCACATCACCCCATAAACACCA | Phe-GAA | tRF-3b | -2.24 | 0.009 |
| tRF-Lys-TTT-001 | CACTGTAAAGCTAAC | Lys-TTT | tRF-5a | -1.96 | 0.001 |
| tRF-Met-CAT-002 | AGTAAGGTCAGCTAAATAAGCTATCGGGCC | Met-CAT | tRF-5c | -1.79 | 0.009 |
| tiRNA-Ser-GCT-001 | GAGAAAGCTCACAAGAACTG | Ser-GCT | tiRNA-5 | -1.59 | 0.005 |
| tiRNA-Tyr-GTA-001 | GGTAAAATGGCTGAGTGAAGCATTGGACTG | Tyr-GTA | tiRNA-5 | -1.58 | 0.008 |
| tRF-Phe-GAA-009 | ATCACCCCATAAACACCA | Phe-GAA | tRF-3a | -1.46 | 0.0127 |
| tRF-Gln-TTG-005 | TAGGATGGGGTGTGAT | Gln-TTG | tRF-5a | -1.37 | 0.0125 |
| tRF-Ser-GCT-005 | GACGAGGTGGCCGA | Ser-GCT | tRF-5a | -1.28 | 0.0412 |
| tRF-Val-TAC-029 | CAGAGTGTAGCTTAACACAAAGCACCCA | Val-TAC | tRF-5c | -1.12 | 0.0411 |
| FC: fold change | | | | | |
